# Temporal changes in auditory adverse event reporting in the U.S. vaccine adverse event reporting system associated with the COVID-19 vaccine rollout: an interrupted time series analysis with negative control evaluation

**DOI:** 10.3389/fpubh.2026.1853253

**Published:** 2026-09-15

**Authors:** Jingyi Wu, Junfeng Huo, Xindan Wang, Juan Wei, Gaiyan Du

**Affiliations:** 1 Public Health Department, Shanxi Bethune Hospital, Shanxi Academy of Medical Sciences, Tongji Shanxi Hospital, Third Hospital of Shanxi Medical University, Taiyuan, China; 2 General Office, Shanxi Provincial Center for Disease Control and Prevention, Taiyuan, China; 3 Immunization Program Department, Taiyuan Center for Disease Control and Prevention, Taiyuan, China

**Keywords:** age stratification, auditory adverse events, COVID-19 vaccine, interrupted time series, reporting propensity, VAERS

## Abstract

**Objectives:**

To assess whether auditory adverse event (AE) reporting changed after the COVID-19 vaccine rollout, and whether any change reflected a true safety signal or a shift in reporting behavior.

**Methods:**

We analyzed U.S. Vaccine Adverse Event Reporting System (VAERS) data from 2018 through 2025 using interrupted time series (ITS) analysis. The COVID-19 vaccine Emergency Use Authorization (EUA), granted in the fourth quarter of 2020, was treated as a time-level ecological intervention. Because VAERS receives reports for all vaccines, we included every report regardless of vaccine type. We then modeled quarterly auditory AE reporting rates before and after the EUA. Sensitivity analyses stratified results by age, fit quasi-Poisson regression models, used dermatologic events as a reporting-propensity sentinel serving much like a negative control, and compared auditory AE proportions across vaccine types after the EUA (COVID-19 versus influenza versus other).

**Results:**

Of 1,347,820 reports, 27,750 involved auditory AEs. Reporting rates rose immediately after the EUA (*β* = 2,317.4, 95% CI 1,079.1–3,555.6; *p* < 0.001). Count-based models incorporating an offset for total reporting confirmed this finding: quasi-Poisson yielded an IRR of 7.14 (95% CI 1.63–31.33; *p* = 0.015), negative binomial an IRR of 5.62 (95% CI 3.29–9.62; *p* < 0.001), and quasi-binomial an OR of 7.36 (95% CI 1.64–33.12; *p* = 0.015). However, the wide confidence intervals indicate that the precise magnitude of the effect was estimated with limited precision. Age stratification revealed clear heterogeneity across age groups. Adults aged 18–64 years exhibited a strong positive effect (*β* = 2,525.2, BH-adjusted *p* = 0.010), as did those aged 65 years and older (*β* = 1,831.9, BH-adjusted *p* = 0.0004), whereas no significant effect was observed among individuals under 18 years (*p* = 0.67). The dermatologic reporting-propensity sentinel demonstrated no meaningful change in absolute reporting counts (IRR 1.09, 95% CI 0.50–2.39; *p* = 0.83). Auditory AEs occurred more frequently in COVID-19 vaccine reports (2.42%) relative to influenza reports (0.88%) and reports for other vaccines (0.84%).

**Conclusion:**

Auditory AE reporting increased after the COVID-19 vaccine EUA. The increase was concentrated in adults and showed up across both rate-based and count-based models, although its magnitude remained imprecisely estimated. It was also specific to COVID-19 vaccine reports. Given the inherent limits of passive surveillance and the non-classical nature of the dermatologic reporting-propensity sentinel, these findings argue for targeted investigation in studies with individual-level data, rather than being read as definitive evidence against reporting bias.

## Background

Auditory symptoms such as tinnitus and hearing loss have been reported following COVID-19 vaccination and have generated substantial public concern. While several case reports and pharmacovigilance studies have suggested a potential association between vaccination and auditory complications, the interpretation of such findings remains challenging due to the inherent limitations of passive surveillance systems (1, 2).

The Vaccine Adverse Event Reporting System (VAERS) is widely used for post-marketing vaccine safety monitoring in the United States (3). Although VAERS plays an essential role in early signal detection, it is subject to several sources of bias, including under-reporting, stimulated reporting, and differential reporting behavior following public health events (4).

During the COVID-19 vaccination campaign, unprecedented media coverage and public attention may have influenced reporting patterns for specific symptoms. As a result, observed increases in certain AE reports may reflect changes in reporting behavior rather than actual changes in incidence.

Interrupted time series (ITS) analysis provides a robust quasi-experimental framework for evaluating temporal changes associated with major interventions. In addition, sensitivity analyses and negative control outcomes can help assess the robustness of observed associations and detect potential reporting artifacts.

Acknowledging the inherent limitations of passive surveillance data, including self-reporting, lack of clinical verification, and susceptibility to reporting biases (e.g., media influence), this study aimed to examine temporal changes in auditory adverse event reporting across the VAERS system surrounding the COVID-19 vaccine rollout. To enhance the robustness of signal detection within this unverified data source, we employed an ITS design augmented by a pre-specified Reporting-Propensity Sentinel Analysis. This approach allows us to distinguish specific temporal patterns for the outcome of interest from non-specific, system-wide changes in reporting behavior, thereby strengthening the interpretability of findings derived from VAERS.

Therefore, using VAERS data from 2018 to 2025, we sought to: (1) quantify the change in auditory AE reporting rates after the EUA, (2) assess heterogeneity across age groups, and (3) critically evaluate whether observed patterns are consistent with a specific signal or a reporting artifact by using dermatologic events as a negative control.

## Methods

### Study design and data source

We conducted an ITS analysis using publicly available data from VAERS, a U.S. national passive surveillance system for post-vaccination AEs. VAERS is a passive, spontaneous reporting system where data are voluntarily submitted and not routinely clinically verified. Consequently, reports are subject to under-reporting, stimulated reporting, and other biases that prevent the calculation of true incidence rates and can create spurious temporal signals. To address these fundamental constraints and improve causal inference, our analysis was explicitly designed to test specificity. The ITS framework controls for underlying pre-intervention trends and seasonality. Crucially, the inclusion of a negative control outcome (dermatologic events) serves as an internal check to detect generalized shifts in reporting that might otherwise be misattributed to a specific vaccine-outcome association. This analysis utilized the final annual VAERS data releases (accessed on March 20, 2026), which encompass reports submitted from 2018 through 2025. We downloaded and merged the VAERSDATA, VAERSVAX, and VAERSSYMPTOMS files for these years. Data cleaning included deduplication using the VAERS_ID field and exclusion of reports with missing key variables (e.g., report date, age).

### Study population and vaccine-level inclusion

The analysis included all VAERS reports submitted between 2018 and 2025, irrespective of the vaccine involved; reports were not restricted to COVID-19 vaccine recipients. This design was dictated by the nature of the exposure: COVID-19 vaccines received their first EUA in December 2020, so restricting the cohort to COVID-19 vaccine reports would leave no pre-EUA baseline against which an ITS could be evaluated. Accordingly, the EUA was treated as an ecological, time-level intervention, and the study evaluates temporal changes in the composition of auditory AE reporting across the entire VAERS system during the COVID-19 vaccination campaign, rather than adverse events attributable to COVID-19 vaccination at the individual level. Vaccine-level information was obtained from the VAERSVAX files using the VAX_TYPE field; vaccine codes beginning with COVID19 (e.g., COVID19, COVID19-2) denote COVID-19 vaccines, codes beginning with ‘FLU’ denote influenza vaccines, and all remaining codes denote other vaccines. To assess whether the observed changes were specific to COVID-19 vaccines, we additionally compared the proportion of reports with an auditory AE among COVID-19, influenza, and other vaccine reports during the post-EUA period (Supplementary Table S3).

### Outcome and exposure definitions

The primary exposure was the EUA of COVID-19 vaccines, operationalized as an intervention starting in 2020 Q4 (December 2020). Auditory AEs were identified if any of the five symptom fields contained predefined keywords related to hearing and balance disorders (e.g., “HEARING LOSS,” “TINNITUS,” “DEAFNESS”; the complete list is available in the Analysis Code). Dermatologic AEs (rash, dermatitis) were identified analogously and were used as a reporting-propensity sentinel (negative-control-like role), intended to detect non-specific changes in reporting behavior. We acknowledge that dermatologic events are recognized reactions to vaccination, for example, cutaneous reactions such as rash and urticaria have been reported after COVID-19 vaccination, and therefore do not satisfy the classic definition of a negative control for confounding (an outcome with no causal association with the exposure). We nevertheless consider dermatologic events informative in this study for three reasons. First, the negative control is used here not to estimate a causal effect of vaccination but to serve as a reporting-propensity sentinel: it is designed to detect a generalized, non-specific shift in reporting behavior (e.g., an across-the-board increase in reporting propensity after the EUA) and to expose denominator-related compositional changes. Second, the direction of any genuine vaccine effect is opposite to what we observed: a true vaccine-induced cutaneous reaction would be expected to increase dermatologic reporting, yet the absolute number of dermatologic reports was unchanged at the EUA (count-based quasi-Poisson IRR 1.09, 95% CI 0.50–2.39; *p* = 0.83) and dermatologic reporting rates declined, a pattern that cannot be explained by a vaccine-associated skin signal. Third, although cutaneous reactions to COVID-19 vaccines have been reported, meta-analyses estimate their pooled incidence at approximately 3–5% of vaccine recipients, with generalized (non-injection-site) reactions substantially less frequent (~1.6%) (5, 6), and these reactions are generally mild and self-limited; they therefore cannot plausibly account for the observed changes in dermatologic reporting, and, importantly, they would be expected to increase, not decrease, dermatologic reporting. Accordingly, the reporting-propensity sentinel remains informative about reporting behavior even though it is not a causal negative control; consistent with this role, it is interpreted only as evidence against a generalized increase in reporting propensity, not as proof of the specificity of the auditory signal.

### Time series construction

Reports were aggregated by calendar quarter, and a continuous integer time index *t* = 1, …, 32 was constructed, where *t* = 1 corresponds to 2018 Q1, *t* = 12 to 2020 Q4, *t* = 17 to 2022 Q1, and *t* = 32 to 2025 Q4. The primary intervention indicator was defined as Intervention1 = 1 if *t* ≥ 12 (COVID-19 vaccine EUA, December 2020) and 0 otherwise. The quarterly auditory AE reporting rate was calculated as (Number of unique reports with an auditory AE/Total number of reports in the quarter) × 100,000. A quarter-to-time-point mapping table is provided in Supplementary Table S1. All figures were generated from this single underlying quarterly dataset and time index, and the placement of every intervention marker was verified against this mapping; the pre-intervention period used for the negative-control parallel-trend assessment (2018 Q1–2020 Q3; *t* = 1–11) is identical to the pre-intervention baseline of the primary model. No quarters in the study period had completely missing data in the raw VAERS extracts; the imputation of zero events refers to quarters where no reports meeting our auditory AE case definition were found, which is a valid representation of the surveillance data. To assess potential bias from differential reporting delays over time, we calculated the lag (in days) between the adverse event onset date (ONSET_DATE) and the report receipt date (RECVDATE) for all auditory AE reports. The median lag periods before and after the COVID-19 vaccine EUA were compared using the Wilcoxon rank-sum test.

### Statistical analysis

Core ITS Analysis: The primary analysis employed a segmented linear regression model on the reporting rates. The quasi-Poisson model was pre-specified as a key sensitivity analysis to account for overdispersion in count data. The primary model was: Reporting Rate ~ Time + Intervention1 + Time Intervention1, where Intervention1 is an indicator (0 before 2020 Q4, 1 after). Given the time-series nature of the data, we used Newey-West standard errors to account for potential autocorrelation in the model residuals, which was formally assessed post-hoc using the Durbin-Watson test.

Age-Stratified Analysis: To explore potential effect modification, reports were categorized into three age groups commonly used in epidemiological and vaccine safety studies: <18 years (children/adolescents), 18–64 years (adults), and ≥65 years (older adults). The core ITS model was fitted separately for each group. To formally test for heterogeneity in the intervention effect across age groups, we compared a pooled model with an age-interaction term to the stratified models using a likelihood ratio test (or Wald test).

### Sensitivity analyses

Quasi-Poisson Regression: Modeled AE counts with an offset for the total reports, to account for overdispersion commonly observed in adverse event count data, where the variance exceeds the mean.

Strict Outcome Definition: Restricted auditory AEs to only “tinnitus” and “hearing loss.”

Reporting-Propensity Sentinel Analysis: Dermatologic events (“rash,” “dermatitis”) were analyzed as a reporting-propensity sentinel (negative-control-like role) using the same ITS framework, including both the rate-based segmented regression and a count-based quasi-Poisson model with an offset for the total number of reports; the count model isolates changes in the absolute number of dermatologic reports from changes in the reporting denominator. We also visually assessed the parallel trends assumption between auditory and dermatologic events during the pre-intervention period (2018 Q1–2020 Q3), which supports the validity of this comparison (Supplementary Figure S3). Because both the auditory and dermatologic rates are scaled to the total number of VAERS reports, we additionally decomposed rate changes into changes in absolute event counts versus changes in total reporting volume (Supplementary Figure S4, Supplementary Table S7).

Model Diagnostics: We performed Durbin-Watson (autocorrelation), Shapiro–Wilk (normality), and Breusch-Pagan (heteroscedasticity) tests on the primary model residuals.

To further differentiate vaccine-specific effects from system-wide changes in reporting behavior, we conducted an additional ITS analysis restricted to reports that did not involve any COVID-19 vaccine (i.e., reports with no VAX_TYPE code beginning with “COVID19”). This analysis repeated the core ITS framework, including the segmented linear regression with Newey-West standard errors, count-based models with an offset for total reporting, age-stratified analysis, and dermatologic reporting-propensity sentinel analysis, on the non-COVID-19 vaccine subset. This approach provides a comparator to assess whether the observed auditory signal is specific to COVID-19 vaccines or reflects broader changes in the VAERS reporting system.

### Intervention timing sensitivity

Because the first COVID-19 EUA was granted on December 11, 2020, near the end of 2020 Q4, the immediate effect of the EUA on reporting may have manifested in the subsequent quarter rather than in 2020 Q4 itself. We therefore re-fitted the primary model with the intervention point placed at 2021 Q1 (*t* = 13) and 2021 Q2 (*t* = 14); results were consistent across specifications (Supplementary Table S6).

We noted that the beta coefficient increased progressively when the intervention was placed later (*t* = 13 and 14) compared with *t* = 12. This pattern is consistent with the delayed manifestation of the EUA in passive surveillance data (e.g., reporting lags and the gradual ramp-up of vaccination coverage) rather than reflecting a progressively stronger biological effect, and does not alter our conclusion that a significant immediate increase occurred regardless of the exact placement of the intervention point.

In an exploratory analysis, we also considered a secondary intervention term for 2022 Q1 to examine potential later changes. However, because this time point lacked a clearly defined event relevant to auditory AE reporting and the resulting model estimates were unstable, we did not include this term in the primary model and it is not considered further in the final conclusions. To address the uncertainty in the exact timing of the primary intervention onset, we instead conducted a sensitivity analysis shifting the intervention to 2021 Q1 and 2021 Q2 (Supplementary Table S6).

*p* values from the three age-stratified comparisons were adjusted using the Benjamini–Hochberg procedure to control the false discovery rate.

All analyses were performed in R (version 4.3.0) using packages including dplyr, lmtest, and ggplot2.

## Results

### Descriptive statistics and overall trends

A total of 1,347,820 VAERS reports from 2018 to 2025 were analyzed, of which 27,750 were identified as auditory AEs (2.06% of all reports). The quarterly auditory AE reporting rate showed moderate fluctuation in the pre-EUA period (2018–2020), followed by a marked increase coinciding with the COVID-19 vaccine rollout in late 2020 (Table 1, Figure 1).

**Table 1:** Descriptive statistics of VAERS reports (2018–2025).

Metric	Value
Total VAERS Reports	1,347,820
Unique Auditory AE Reports	27,750
Overall Auditory AE Proportion	2.06%
Unique Dermatologic AE Reports	101,022
Pre-EUA (2018 Q1–2020 Q3) Average Quarterly Reporting Rate (per 100 k)	963.0
Post-EUA (2020 Q4–2025 Q4) Average Quarterly Reporting Rate (per 100 k)	1,637.9

Data source: Vaccine Adverse Event Reporting System (VAERS), 2018–2025. The reporting rate is calculated as the number of unique auditory AE reports per 100,000 total reports in a given quarter. EUA = Emergency Use Authorization. Dermatologic events served as the negative control outcome.

**Figure 1:**
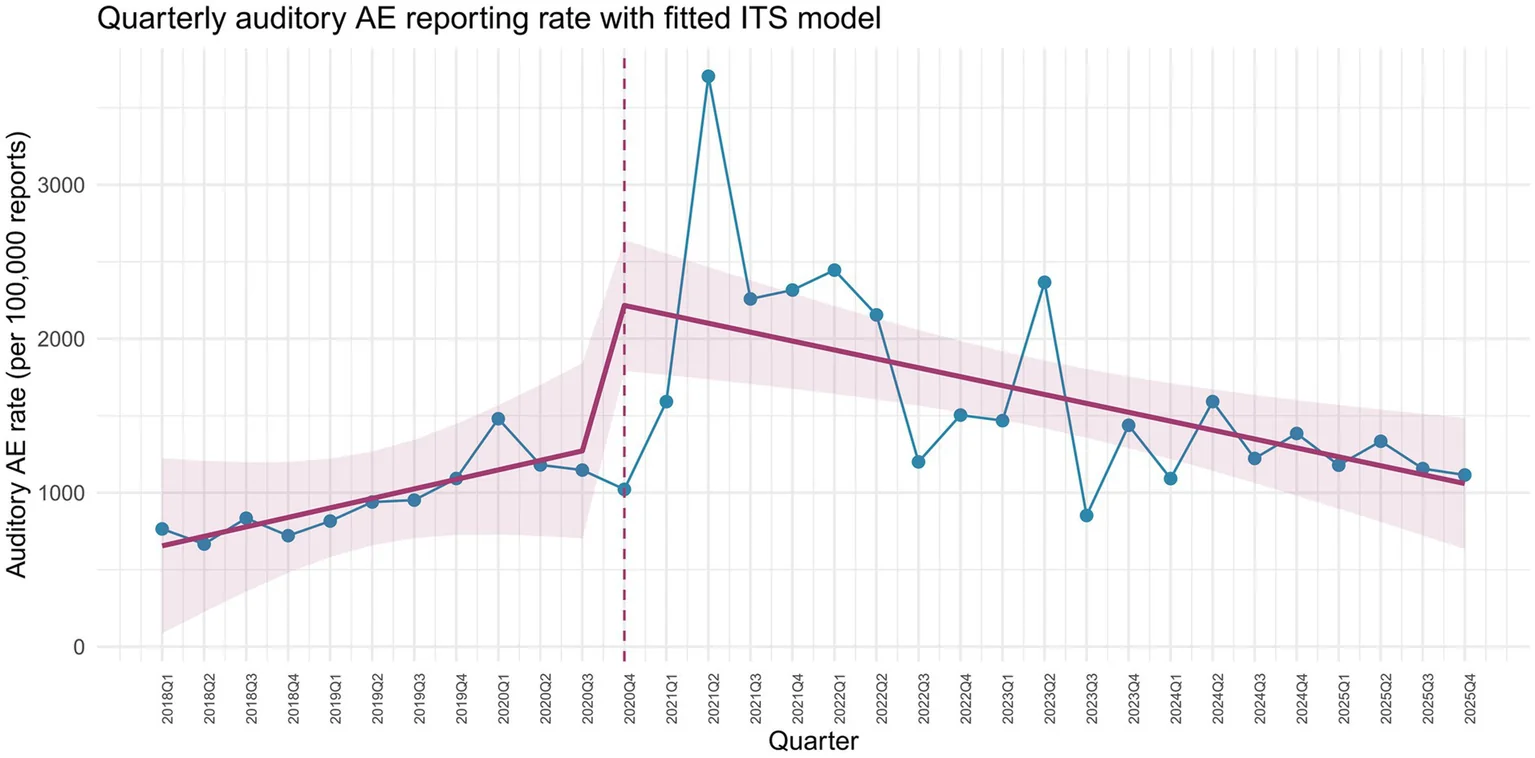
Quarterly reporting rates for auditory AEs (2018–2025), with a vertical dashed line indicating the primary intervention at *t* = 12 (2020 Q4), placed using the same integer time index as the primary ITS model. The shaded area represents the 95% confidence interval around the fitted trend line from the primary ITS model; residuals deviated from normality (Shapiro–Wilk *p* = 0.004).

### Core interrupted time series analysis

The primary ITS model indicated a significant immediate level increase in the auditory AE reporting rate following the EUA in 2020 Q4 (*β* = 2,317.4, CI 1,079.1–3,555.6; *p* < 0.001), accompanied by a deceleration in the post-intervention trend (*β* = −119.5, 95% CI −171.9 to −67.2; *p* < 0.001; Table 2). Because the first EUA occurred near the end of 2020 Q4, we verified that the immediate increase remained significant when the intervention was placed at 2021 Q1 or 2021 Q2 (Supplementary Table S6).

**Table 2:** Coefficients from the primary interrupted time series model.

Term	Estimate (*β*)	Std. Error	*p*-value	Interpretation
(Intercept)	593.0	39.1	<0.001	Baseline level (2018 Q1)
Time	61.7	7.6	<0.001	Pre-intervention trend per quarter
Intervention1 (2020 Q4)	2,317.4	631.8	0.001	Immediate level change post-EUA
Time: Intervention1	−119.5	24.2	<0.001	Change in trend post-EUA

Results from the primary segmented linear regression model. Newey-West standard errors (lag = 3) were used to account for autocorrelation. *R*^2^ = 0.48. EUA = Emergency Use Authorization. Significance codes: ^***^p < 0.001, ^*^p < 0.05.

### Count-based models

Because the outcome is a count of auditory AE reports relative to a total, we fitted segmented count models with an offset for the log-transformed total number of reports and treated them as co-primary alongside the rate-based model. The immediate increase following the EUA was confirmed in all count models: quasi-Poisson (IRR 7.14 (1.63–31.33) *p* = 0.015), negative binomial (IRR 5.62, 95% CI 3.29–9.62; *p* < 0.001), and quasi-binomial regression on the proportion of reports involving an auditory AE (OR 7.36, 95% CI 1.64–33.12; *p* = 0.015; Supplementary Table S2). Confidence intervals were wide, indicating that the precise magnitude of the effect was estimated with limited precision.

### Age-stratified analysis

The intervention effect exhibited marked age heterogeneity (Figure 2, Supplementary Table S5). Strong positive immediate effects were observed in adults aged 18–64 years (*β* = 2,525.2, 95% CI 828.2–4,222.2; BH-adjusted *p* = 0.010) and in those ≥65 years (*β* = 1,831.9, 95% CI 1,016.3–2,647.4; BH-adjusted *p* = 0.0004). No significant immediate effect was found in individuals <18 years (*β* = −166.9, 95% CI −915.6 to 581.9; *p* = 0.67; Supplementary Figure S2). A formal test for interaction between age group and the intervention effect was significant (likelihood ratio test *p* < 0.001), confirming statistically heterogeneous effects across age groups (Supplementary Table S5).

**Figure 2:**
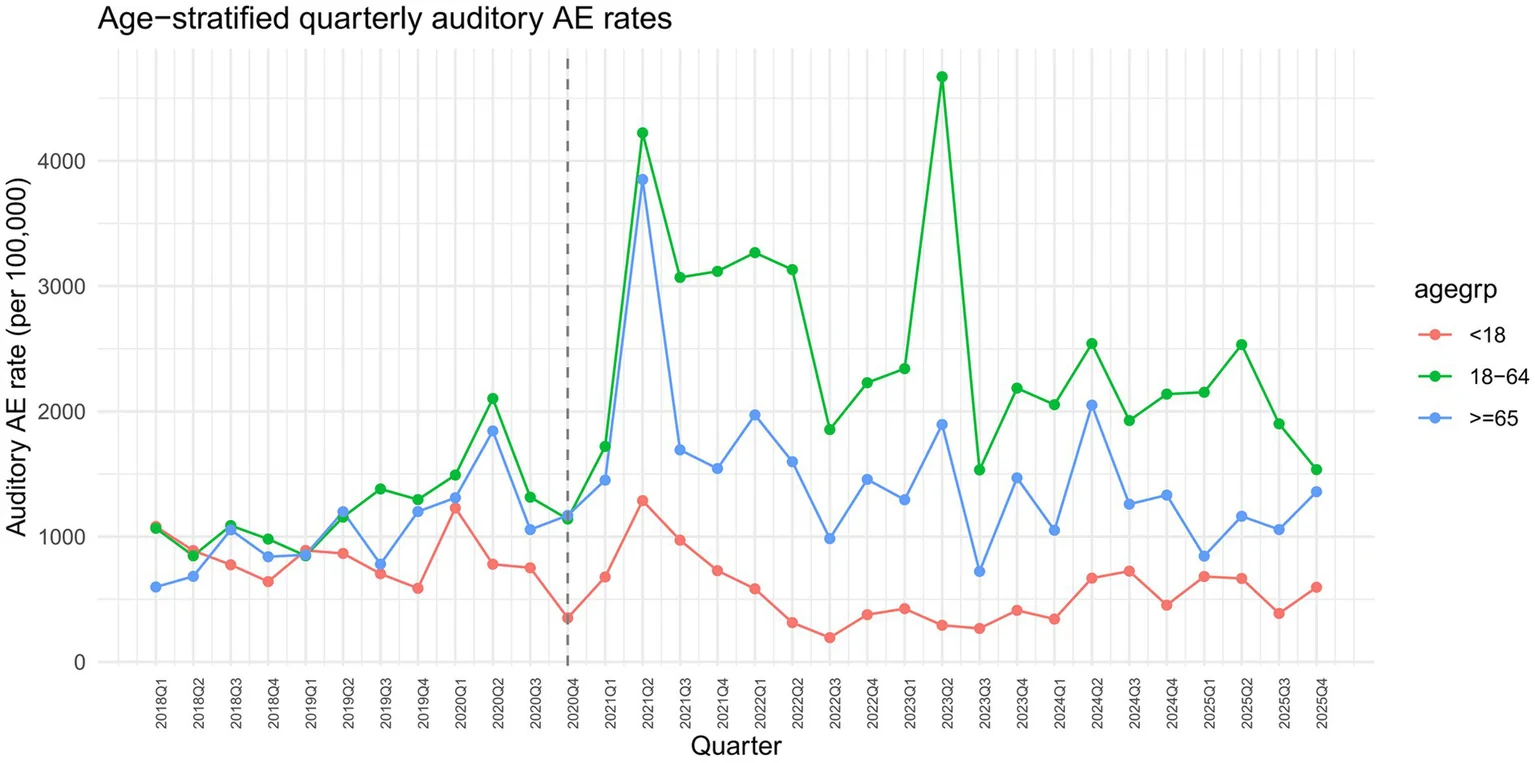
Age-stratified trends in auditory AE reporting rates (2018–2025). The vertical dashed line indicates the COVID-19 vaccine EUA (2020 Q4). Specific BH-adjusted *p*-values (18–64 years: 0.010; ≥65 years: <0.001; <18 years: 0.67) are shown directly adjacent to each age-group line in the figure.

### Assessment of reporting delays

The median reporting lag for auditory AEs was 13 days (IQR: 3–57) in the pre-EUA period and increased to 26 days (IQR: 6–112) in the post-EUA period (Wilcoxon test *p* < 0.001). This indicates that reports were submitted more slowly after the vaccine rollout. However, in an ITS framework, such a systematic change in lag is unlikely to create a spurious abrupt level shift at the exact intervention point; it would more likely manifest as a gradual trend change. Therefore, the observed significant immediate increase in reporting rates is not readily attributable to this delay pattern.

### Reporting-propensity sentinel analysis

Because dermatologic events such as rash and dermatitis may themselves be associated with COVID-19 vaccination, they do not meet the strict definition of a causal negative control outcome. We therefore used dermatologic events as a reporting-propensity sentinel (negative-control-like role), intended to assess whether a broad, non-specific change in reporting propensity occurred around the COVID-19 vaccine rollout. This analysis was not intended to establish that dermatologic events were causally unrelated to vaccination or to exclude all forms of reporting bias.

Analysis of dermatologic events (rash, dermatitis) revealed a marked decline in reporting rates following the EUA (*β* = −9,472.3, *p* = 0.081, post-EUA trend change *β* = +564.2/quarter, *p* = 0.03; Figure 3, Supplementary Table S4). However, this rate decline was fully explained by denominator expansion: the absolute number of dermatologic reports grew from 15,642 (2018 Q1–2020 Q3) to 83,176 (2021 Q1–2025 Q4), a 5.3-fold increase that was smaller than the 9.7-fold increase in total VAERS reporting, and a count-based quasi-Poisson model with an offset for total reports showed no significant change in dermatologic reporting at the EUA (IRR 1.09, 95% CI 0.50–2.39; *p* = 0.83; Supplementary Table S4b). The dermatologic rate decline therefore reflects expansion of the total-reporting denominator rather than a change in dermatologic reporting behavior. Visual inspection of pre-intervention trends confirmed that auditory and dermatologic events followed parallel trajectories prior to the EUA (Supplementary Figure S3). To supplement this visual assessment, we separately regressed the unscaled quarterly rates on time during the pre-EUA period (*t* = 1–11). The estimated pre-intervention slopes were 61.7 per 100,000 per quarter for auditory events and −59.0 per 100,000 per quarter for dermatologic events (when scaled comparably for the figure); this similar magnitude of change supports the parallel trends assumption underlying the negative-control comparison. (The opposite signs reflect the opposite baseline directions of the two outcomes, not a violation of parallelism.). Taken together, these analyses indicate that the auditory signal did not arise as part of a generalized, system-wide increase in reporting propensity; however, they do not, by themselves, establish the specificity of the auditory signal or exclude denominator-related and compositional artifacts. The absolute-count analyses (Supplementary Figure S4, Supplementary Table S7) address this directly: auditory AE counts increased 22.8-fold (1,155–26,331), far exceeding the 9.7-fold growth in total reporting, indicating that the auditory rate increase was not an artifact of a shrinking denominator.

**Figure 3:**
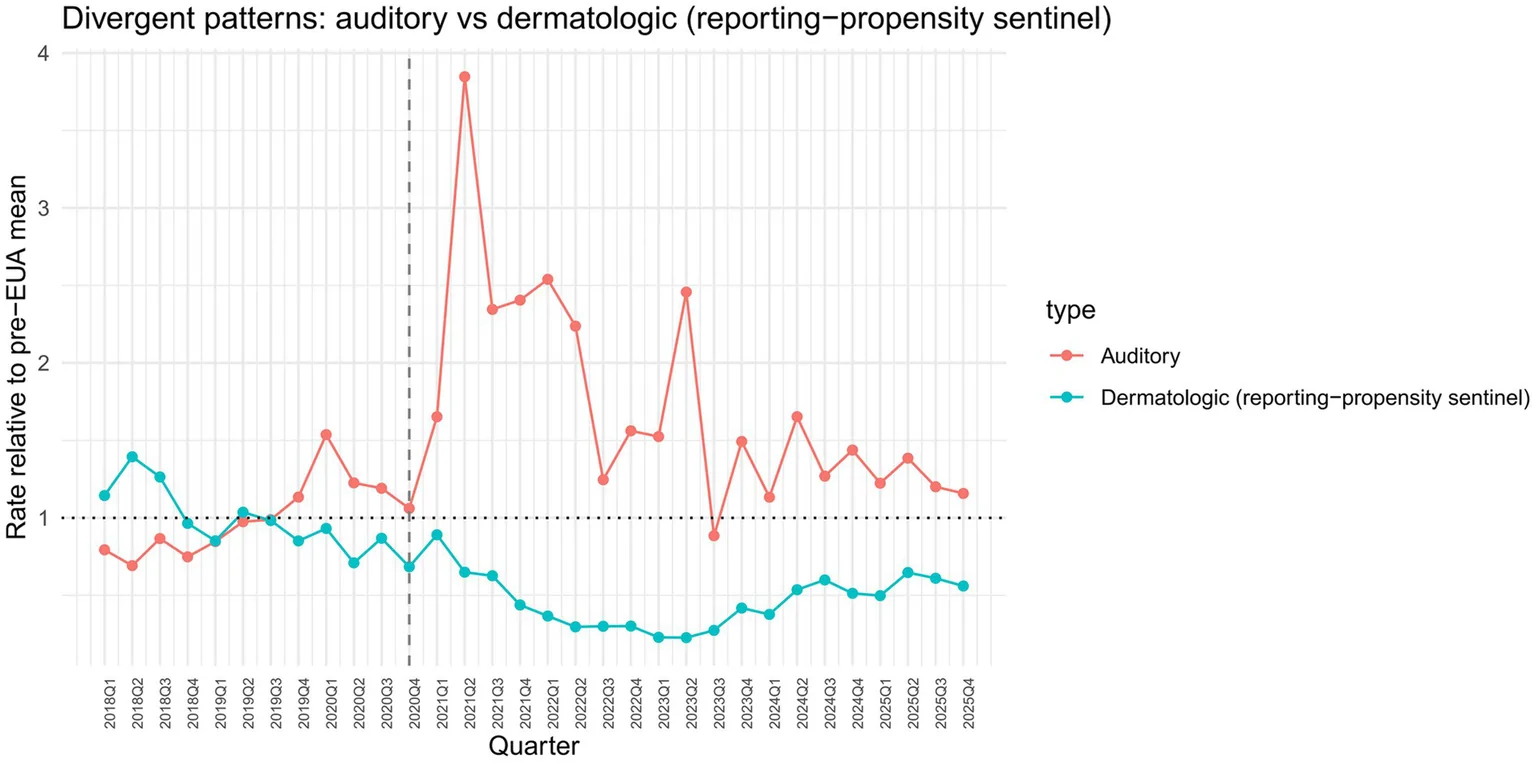
Divergent trends in reporting rates between auditory adverse events and dermatologic events used as a reporting-propensity sentinel following the COVID-19 vaccine EUA. The dashed vertical line indicates the EUA (2020 Q4; *t* = 12), placed consistently with Figures 1, 2. While auditory AE reports increased sharply, reports of dermatologic events (rash, dermatitis) showed an immediate and sustained decrease, contradicting the expectation of a non-specific, generalized increase in reporting.

### Vaccine-type analysis

During the post-EUA period (2020 Q4–2025 Q4), the proportion of reports involving an auditory AE was 2.42% among COVID-19 vaccine reports (24,974/1,032,251), compared with 0.88% among influenza vaccine reports (347/39,661) and 0.84% among reports of other vaccines (1,274/152,083; overall chi-square *p* < 0.001; COVID-19 vs. other vaccines *p* < 0.001; influenza vs. other vaccines *p* = 0.47; Supplementary Table S3, Figure 4). The auditory signal was therefore specifically enriched in COVID-19 vaccine reports, rather than distributed uniformly across all vaccine types.

**Figure 4:**
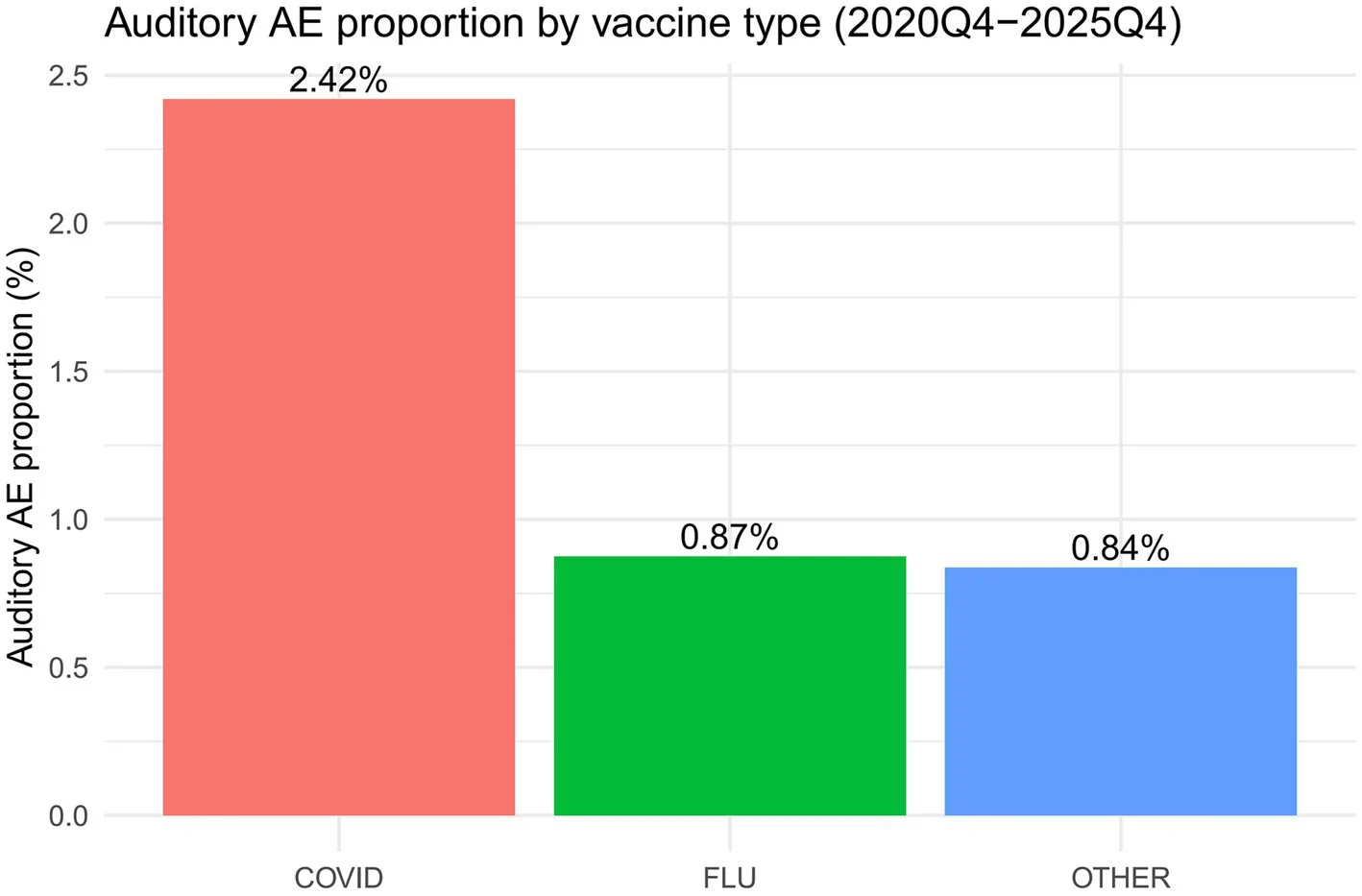
Auditory AE proportion by vaccine type during the post-EUA period (2020 Q4–2025 Q4). Proportions are shown for COVID-19 vaccine reports (2.42%), influenza vaccine reports (0.88%), and other vaccine reports (0.84%). The overall difference across vaccine types was statistically significant (chi-square *p* < 0.001). Error bars indicate 95% confidence intervals for the proportions.

Because COVID-19 vaccines were not available before the EUA, the vaccine-type comparison was restricted to the post-EUA period (2020 Q4–2025 Q4), during which 26,595 of the 27,750 auditory AE reports occurred; the remaining 1,155 auditory AE reports, submitted before the EUA (2018 Q1–2020 Q3), could not be attributed to COVID-19 vaccination and were therefore excluded from this comparison.

### Sensitivity and supplementary analyses

Count-based models: The quasi-Poisson, negative binomial, and quasi-binomial results are presented as co-primary analyses in the Results (Count-based models). In an exploratory sensitivity analysis, applying Newey–West (HAC) standard errors to the quasi-Poisson model produced unstable estimates with only 32 quarterly observations and very wide confidence intervals; these are therefore not relied upon, and the absence of explicit autocorrelation terms in the count models should be acknowledged as a limitation.

Strict Outcome Definition: Restricting auditory AEs to “tinnitus” and “hearing loss” (*N* = 19,490), the immediate level increase post-EUA remained significant (*β* = 1,870.8, 95% CI 853.9–2,887.6; *p* = 0.001).

In a sensitivity analysis restricted to non-COVID-19 vaccine reports (*n* = 315,568; auditory AE = 2,776), the immediate increase in auditory AE reporting remained statistically significant but was substantially attenuated compared with the primary analysis. The segmented linear regression yielded a beta coefficient of 702.4 (95% CI: 354.3–1,050.5; *p* < 0.001), representing approximately 30% of the effect observed in the primary analysis (*β* = 2,317.4). Count-based models with an offset for total reporting confirmed this attenuated signal: quasi-Poisson IRR = 2.24 (95% CI: 1.53–3.29; *p* < 0.001) and negative binomial IRR = 2.19 (95% CI: 1.51–3.18; *p* < 0.001). Age-stratified analysis within the non-COVID subset revealed significant heterogeneity: the <18 years group showed a decline (*β* = −404.1, BH-adjusted *p* = 0.001), while the 18–64 years group (*β* = 1,391.7, BH-adjusted *p* < 0.001) and ≥65 years group (*β* = 1,351.7, BH-adjusted *p* < 0.001) showed increases. Notably, the dermatologic reporting-propensity sentinel in the non-COVID analysis showed a significant decrease in absolute counts (IRR = 0.46, 95% CI: 0.34–0.62; *p* < 0.001), further supporting that the dermatologic rate decline was not due to increased reporting. These results are presented in Supplementary Tables S8–S11.

Model Diagnostics: Residuals of the primary model showed no significant autocorrelation (Durbin–Watson statistic = 2.01, *p* = 0.29) but deviated from normality (Shapiro–Wilk *p* = 0.004) and indicated heteroscedasticity (Breusch–Pagan *p* = 0.013). To assess the robustness of our inferences to these violations, we re-fitted the primary ITS model using heteroscedasticity-consistent (HC3) and Newey–West standard errors; the immediate level increase post-EUA remained significant with both (HC3-adjusted *p* = 0.005; Newey–West *p* = 0.001; Supplementary Figure S1).

## Discussion

### Principal findings

This ITS analysis of VAERS data identified a temporal increase in auditory AE reporting rates following the EUA of COVID-19 vaccines, manifesting as an immediate level change rather than a sustained trend shift. As an analysis of passive surveillance data, our findings must be interpreted as changes in reporting patterns rather than as measures of causal risk or true incidence. The pattern was heterogeneous across age groups, with a more pronounced increase among adults aged 18–64 years and no significant change among individuals <18 years. In addition, the absence of a similar increase in the dermatologic reporting-propensity sentinel, together with the absence of evidence for a generalized increase in reporting propensity, suggests that the observed pattern is not readily explained by a single, uniform reporting mechanism. However, these observations should be interpreted with caution given the inherent limitations of passive surveillance data.

### Interpretative context and causal inference

It is crucial to reiterate that findings from passive surveillance like VAERS cannot establish causality. The signal identified here necessitates confirmation through well-designed analytical epidemiological studies (e.g., cohort or case–control studies) with individual-level data and adjustment for confounding. The following interpretations are therefore framed within the context of generating hypotheses for further investigation, rather than proving definitive biological mechanisms.

### Interpretation and implications

The age-stratified findings challenge a singular explanation of non-specific reporting bias (4, 7, 8). In interpreting these heterogeneous effects, differential reporting behaviors across age groups must be considered as a primary potential explanation. Caregivers may prioritize reporting immediate, systemic reactions (e.g., fever) over subtler auditory symptoms in children, whereas adults are capable of self-reporting such nuanced sensations (9–11). Furthermore, during the mass vaccination campaign, heightened attention to severe pediatric conditions like myocarditis may have led to the under-reporting or misattribution of auditory symptoms in the <18 years group (12, 13); Beyond reporting artifacts, other plausible hypotheses include immunological or developmental differences, as the auditory system and immune responses vary with age (14, 15), and confounding by co-administration with other childhood vaccines.

The observed age heterogeneity indicates that vaccine safety signals are not uniform and must be investigated with careful attention to effect modifiers like age, highlighting the inadequacy of “one-size-fits-all” approaches. The contrast between auditory and dermatologic reporting patterns provides some evidence against a uniform, system-wide increase in reporting propensity. A generalized increase in reporting would be expected to affect multiple commonly reported adverse events in a similar direction, whereas dermatologic events did not show a corresponding increase in absolute reporting counts. However, because dermatologic events may themselves be associated with COVID-19 vaccination and because reporting rates are influenced by changes in the overall VAERS reporting denominator, this comparison should not be interpreted as evidence of causal specificity. However, as discussed below, the compositional (denominator) limitation of rate-based comparisons means that this divergence does not, by itself, establish the specificity of the auditory signal, and hypotheses such as “reporting competition” or “attention dilution” remain speculative.

The non-COVID-19 vaccine sensitivity analysis provides additional insight into the specificity of the observed auditory signal. While a statistically significant increase remained in the non-COVID subset, the effect size was markedly attenuated, the beta coefficient (702.4) was only about 30% of that in the primary analysis (2,317.4), and the IRR decreased from 7.14 to 2.24. This pattern suggests that while a modest degree of system-wide reporting change may have occurred during the pandemic (e.g., heightened public awareness, facilitated reporting channels), the auditory signal is predominantly driven by COVID-19 vaccine reports. Furthermore, the unexpected finding of a significant decline in non-COVID dermatologic reports (IRR = 0.46) stands in contrast to the unchanged dermatologic counts in the primary analysis, likely reflecting denominator-related compositional changes in the non-COVID reporting pool. Taken together, these results indicate that the auditory signal is largely, though not exclusively, specific to COVID-19 vaccines.

Nevertheless, the absolute-count analysis showed that auditory AE reports increased 22.8-fold (from 1,155 to 26,331) while total reporting increased only 9.7-fold and dermatologic reporting 5.3-fold; the auditory signal thus emerged against a backdrop in which the overall reporting denominator expanded far less than auditory counts, which argues against the increase being a mere artifact of a shrinking denominator or of increased vigilance (16–19).

The negative control analysis addresses one specific concern: a broad, non-specific increase in reporting propensity. It does not, however, exclude denominator-related or compositional changes, and it should not be read as direct evidence against reporting bias more generally.

A key consideration is the consistency of the findings across model specifications. After correction of the outcome ascertainment, the immediate increase in auditory AE reporting was statistically significant in the rate-based segmented linear regression (*β* = 2,317.4, *p* < 0.001) and in all count-based models with an offset for total reporting, quasi-Poisson (IRR 7.14 (1.63–31.33) *p* = 0.015), negative binomial (IRR 5.62, 95% CI 3.29–9.62; *p* < 0.001), and quasi-binomial (OR 7.36, 95% CI 1.64–33.12; *p* = 0.015). The direction of the effect was therefore consistent across model families. At the same time, the wide confidence intervals indicate that the precise magnitude of the effect is estimated with limited precision, reflecting the modest number of quarterly observations (*n* = 32), the substantial overdispersion, and the influence of a few high-reporting quarters. Because inference from surveillance data can depend on model specification, we emphasize the temporal signal itself rather than the exact effect size, and we caution against over-interpreting the quantitative strength of the association within the VAERS data framework.

Because a genuine vaccine-induced cutaneous reaction would be expected to increase, rather than decrease, dermatologic reporting, and because the absolute number of dermatologic reports was unchanged at the EUA (IRR 1.09, 95% CI 0.50–2.39; *p* = 0.83), the observed decline in dermatologic reporting rates cannot be explained by a true vaccine–skin effect.

### Compositional (denominator) limitation

Because all reporting rates in this study are scaled to the total number of VAERS reports in each quarter, a sharp expansion in the volume of total reporting, as occurred during the COVID-19 vaccination campaign, will, by itself, lower the reported rate of any adverse-event category whose absolute reporting volume does not increase proportionally, and will simultaneously raise the rate of categories whose absolute volume grows faster than the denominator, even in the absence of any causal relationship. This is precisely what we observed for dermatologic events. Their absolute reporting volume grew 5.3-fold (from 15,642 to 83,176 reports) between the pre-EUA period (2018 Q1–2020 Q3) and 2021 Q1–2025 Q4, whereas total reporting grew 9.7-fold over the same period; arithmetically, this differential growth alone would reduce the dermatologic reporting rate to approximately 55% of its pre-EUA level (5.3 ÷ 9.7), a relative decline of roughly 45%. Consistent with this, a count-based quasi-Poisson model with an offset for total reports showed no significant change in dermatologic reporting at the EUA (IRR 1.09, 95% CI 0.50–2.39; *p* = 0.83), confirming that the dermatologic rate decline reflects expansion of the reporting denominator rather than a change in dermatologic reporting behavior. Consequently, the denominator expansion is sufficient to account for the observed decline in dermatologic reporting rates and does not require the ‘reporting competition’ or ‘attention dilution’ hypotheses. The denominator effect cannot, however, explain the auditory increase, because the absolute number of auditory AE reports grew 22.8-fold (from 1,155 to 26,331), far exceeding the 9.7-fold expansion of the denominator; the observed auditory rate increase is therefore inconsistent with being explained solely by expansion of the reporting denominator.

### Strengths and limitations

Strengths include the use of an ITS design, multiple sensitivity and stratification analyses, and a reporting-propensity sentinel to assess broad, non-specific changes in reporting behavior.

*Key limitations persist*: The most significant limitation of this study stems from its use of VAERS data, which are self-reported, unverified, and affected by reporting biases that we cannot directly measure or adjust for. These include under-reporting, media-stimulated reporting, and the ‘healthy vaccinee’ effect. Therefore, our results reflect temporal changes in reporting rates and cannot establish causation or quantify true risk. An additional limitation is that our analysis did not account for the number of vaccine doses administered per individual. During the COVID-19 vaccination campaign, the widespread administration of multiple doses (e.g., primary series and boosters) may have increased the opportunity for adverse event reporting per individual. This could have contributed to the observed increase in absolute counts, as a single individual could have filed multiple reports over time. While the VAERS public-use data do not allow us to reliably link reports to unique individuals across multiple vaccinations, this represents an important source of bias that should be considered when interpreting the magnitude of the increase. Furthermore, the age-dependent rollout of COVID-19 vaccines, with older adults prioritized for earlier vaccination, may have confounded our age-stratified results. The observed immediate increase in the ≥65 and 18–64 age groups, and the lack thereof in the <18 group, could reflect the phased introduction of vaccines to these populations rather than, or in addition to, differential biological susceptibility or reporting propensity. As our analysis did not incorporate quarterly, age-specific vaccination coverage data as a time-varying covariate, we cannot disentangle the effect of vaccine availability from the effect of vaccination itself on reporting rates. This limitation underscores that the age-heterogeneity should be interpreted as a hypothesis-generating finding. However, our study design incorporated strategies to assess the robustness of the signal within these constraints. The ITS design controlled for pre-existing trends. The negative control analysis provided a test for one specific type of artifact, a generalized increase in reporting propensity, and this was not observed (dermatologic counts were unchanged). It does not exclude denominator or compositional effects. Nonetheless, the absolute-count analysis showed that auditory AE reports increased far more (22.8-fold) than total reporting (9.7-fold), which argues against the auditory rate increase being a mere denominator artifact. Ultimately, analyses of passive surveillance data like VAERS are best suited for hypothesis generation. Our findings, in particular the age-heterogeneous pattern and the disproportionate increase in absolute auditory AE counts, highlight a potential safety signal that warrants formal investigation in studies with individual-level data, clinical validation of outcomes, and controlled comparisons, such as cohort or case–control designs.

### Implications for public health surveillance and practice

The findings of this study, while derived from passive surveillance and requiring confirmation, offer several actionable insights for enhancing vaccine safety systems and clinical practice. First, to improve signal specificity in pharmacovigilance, national monitoring programs could consider implementing targeted signal reviews for auditory symptoms reported in adults, particularly those aged 18–64 years where the signal was strongest. This could involve dedicated medical review of cases or refining diagnostic coding within reporting systems. Furthermore, incorporating reporting-propensity sentinel analyses as a complementary sensitivity check could help assess broad, non-specific reporting changes in future safety evaluations. Second, for clinical and public communication, health authorities may consider including tinnitus and hearing changes in post-vaccination information sheets as rare symptoms warranting medical attention if they occur. This should be communicated alongside clear data on the extremely low absolute risk, balancing awareness with the imperative to prevent undue vaccine hesitancy. Finally, this analysis generates a prioritized hypothesis for further investigation. Subsequent research should employ study designs with individual-level data and clinical validation, such as retrospective cohort or case–control studies using electronic health records (EHRs), to quantify the absolute risk and establish temporal association more robustly. Such studies should be particularly focused on the adult population to verify the age-stratified pattern observed here.

## Conclusion

In summary, this analysis identifies a temporal signal of increased auditory AE reports in VAERS associated with the COVID-19 vaccine rollout, with the signal specifically concentrated in COVID-19 vaccine reports and in adults, and consistently detected across rate-based and count-based model specifications; however, the precise magnitude of the effect is estimated with limited precision, reflecting the modest number of quarterly observations and the substantial overdispersion, and should therefore be interpreted cautiously. The age-heterogeneous pattern and the disproportionate increase in absolute auditory AE counts relative to total reporting support the interpretation that the signal is not a simple denominator artifact; however, given the inherent limitations of passive surveillance data, these findings should be interpreted as hypothesis-generating and warrant targeted investigation in studies with individual-level data. Although derived from U.S. surveillance data, the methodological approach and the generated hypothesis have global relevance. VAERS is one of the largest systems of its kind, and signals identified within it often prompt investigations in other regions. Our findings suggest that national pharmacovigilance programs, particularly in settings with high COVID-19 vaccine coverage in adults, could consider targeted reviews of auditory AE reports. The age-heterogeneous pattern further highlights the universal need for age-stratified safety monitoring. While these unverified data cannot prove causation, the results generate a hypothesis that merits targeted investigation in more robust epidemiological studies, with particular attention to the adult population. Such research is crucial for informing vaccine safety monitoring and clinical guidance.

## Data Availability

The original contributions presented in the study are included in the article/Supplementary material, further inquiries can be directed to the corresponding author.
