# Supplementary material for: Temporal changes in auditory adverse event reporting in the U.S. vaccine adverse event reporting system associated with the COVID-19 vaccine rollout: an interrupted time series analysis with negative control evaluation
Source: Front Public Health. 2026 Sep 15;14:1853253. doi: 10.3389/fpubh.2026.1853253 (PMC13619909; doi:10.3389/fpubh.2026.1853253)
Supplement: Supplementary file 1 [file Data_Sheet_1.DOCX]

**Supplementary Table 1. Quarter-to-Time-Point Mapping**

| **Time point (t)** | **Year** | **Quarter** | **Calendar quarter** | **Intervention1** | **Intervention2** |
| --- | --- | --- | --- | --- | --- |
| 1 | 2018 | 1 | 2018Q1 | 0 | 0 |
| 2 | 2018 | 2 | 2018Q2 | 0 | 0 |
| 3 | 2018 | 3 | 2018Q3 | 0 | 0 |
| 4 | 2018 | 4 | 2018Q4 | 0 | 0 |
| 5 | 2019 | 1 | 2019Q1 | 0 | 0 |
| 6 | 2019 | 2 | 2019Q2 | 0 | 0 |
| 7 | 2019 | 3 | 2019Q3 | 0 | 0 |
| 8 | 2019 | 4 | 2019Q4 | 0 | 0 |
| 9 | 2020 | 1 | 2020Q1 | 0 | 0 |
| 10 | 2020 | 2 | 2020Q2 | 0 | 0 |
| 11 | 2020 | 3 | 2020Q3 | 0 | 0 |
| 12 | 2020 | 4 | 2020Q4 | 1 | 0 |
| 13 | 2021 | 1 | 2021Q1 | 1 | 0 |
| 14 | 2021 | 2 | 2021Q2 | 1 | 0 |
| 15 | 2021 | 3 | 2021Q3 | 1 | 0 |
| 16 | 2021 | 4 | 2021Q4 | 1 | 0 |
| 17 | 2022 | 1 | 2022Q1 | 1 | 1 |
| 18 | 2022 | 2 | 2022Q2 | 1 | 1 |
| 19 | 2022 | 3 | 2022Q3 | 1 | 1 |
| 20 | 2022 | 4 | 2022Q4 | 1 | 1 |
| 21 | 2023 | 1 | 2023Q1 | 1 | 1 |
| 22 | 2023 | 2 | 2023Q2 | 1 | 1 |
| 23 | 2023 | 3 | 2023Q3 | 1 | 1 |
| 24 | 2023 | 4 | 2023Q4 | 1 | 1 |
| 25 | 2024 | 1 | 2024Q1 | 1 | 1 |
| 26 | 2024 | 2 | 2024Q2 | 1 | 1 |
| 27 | 2024 | 3 | 2024Q3 | 1 | 1 |
| 28 | 2024 | 4 | 2024Q4 | 1 | 1 |
| 29 | 2025 | 1 | 2025Q1 | 1 | 1 |
| 30 | 2025 | 2 | 2025Q2 | 1 | 1 |
| 31 | 2025 | 3 | 2025Q3 | 1 | 1 |
| 32 | 2025 | 4 | 2025Q4 | 1 | 1 |

*Note: t = 1 corresponds to 2018 Q1; t = 12 to 2020 Q4 (COVID-19 vaccine EUA); t = 17 to 2022 Q1; t = 32 to 2025 Q4. Intervention1 = 1 if t ≥ 12; Intervention2 = 1 if t ≥ 17.*

**Supplementary Table 2. Count-Based Segmented Models**

| **Model** | **Offset** | **Effect** | **Estimate** | **95% CI** | **p** |
| --- | --- | --- | --- | --- | --- |
| Quasi-Poisson | log(total reports) | IRR | 7.14 | 1.63–31.33 | 0.015 |
| Negative binomial | log(total reports) | IRR | 5.62 | 3.29–9.62 | <0.001 |
| Quasi-binomial | — | OR | 7.36 | 1.64–33.12 | 0.015 |

*Note: Count-based segmented regression with an offset for the log-transformed total number of reports. IRR = incidence rate ratio; OR = odds ratio. The estimate represents the immediate change following the EUA.*

**Supplementary Table 3. Vaccine-Type Comparison of Auditory AE Proportions (Post-EUA Period)**

| **Vaccine type** | **Reports (n)** | **Auditory AE (n)** | **Proportion (%)** |
| --- | --- | --- | --- |
| COVID-19 | 1,032,251 | 24,974 | 2.42 |
| Influenza | 39,661 | 347 | 0.88 |
| Other | 152,083 | 1,274 | 0.84 |

*Note: Post-EUA period (2020 Q4–2025 Q4). Overall chi-square p < 0.001; COVID-19 vs. other vaccines p < 0.001; influenza vs. other vaccines p = 0.47.*

**Supplementary Table 4. Reporting-Propensity Sentinel (Dermatologic) Outcome — Rate-Based ITS Model**

| **Term** | **Estimate (β)** | **p-value** | **Interpretation** |
| --- | --- | --- | --- |
| (Intercept) | 16004.9 | <0.001 | Baseline dermatologic reporting rate (2018 Q1) |
| Time (per quarter) | −590.2 | <0.001 | Pre-EUA trend |
| Intervention1 (2020 Q4) | −9472.3 | 0.081 | Immediate level change (borderline) |
| Time × Intervention1 | +564.2 | 0.032 | Post-EUA trend change |

*Note: Segmented linear regression on the quarterly dermatologic reporting rate (per 100,000 reports) with Newey–West standard errors (lag = 3).*

**Supplementary Table 4b. Reporting-Propensity Sentinel — Count-Based (Quasi-Poisson) Model**

| **Effect (IRR)** | **95% CI** | **p-value** |
| --- | --- | --- |
| 1.09 | 0.50–2.39 | 0.83 |

*Note: Count-based quasi-Poisson model with an offset for the total number of reports; the IRR represents the immediate change in the absolute number of dermatologic reports at the EUA. IRR = incidence rate ratio.*

**Supplementary Table 5. Age-Stratified Interrupted Time Series Results for the EUA Intervention Effect**

| **Age group** | **β** | **95% CI** | **Raw p** | **BH-adjusted p** | **Model R²** |
| --- | --- | --- | --- | --- | --- |
| 18–64 years | 2525.2 | 828.2–4222.2 | 0.007 | 0.010 | 0.45 |
| ≥65 years | 1831.9 | 1016.3–2647.4 | <0.001 | 0.0004 | 0.32 |
| <18 years | −166.9 | −915.6 to 581.9 | 0.67 | 0.67 | 0.29 |

*Note: Segmented linear regression with Newey–West standard errors. p-values were adjusted using the Benjamini–Hochberg (BH) method for three age-group comparisons.*

**Supplementary Table 6. Intervention Timing Sensitivity**

| **Intervention point** | **β** | **95% CI** | **p-value** |
| --- | --- | --- | --- |
| 2020 Q4 (t = 12) | 2317.4 | 1079.1–3555.6 | 0.001 |
| 2021 Q1 (t = 13) | 2749.6 | 1896.2–3603.0 | <0.001 |
| 2021 Q2 (t = 14) | 3163.5 | 1970.2–4356.7 | <0.001 |

*Note: Rate-based linear model with Newey–West standard errors; the primary intervention was placed at the indicated quarter.*

**Supplementary Table 7. Absolute Report Counts by Study Period**

| **Study period** | **Total reports** | **Auditory AE** | **Dermatologic** |
| --- | --- | --- | --- |
| Pre-EUA (2018 Q1–2020 Q3) | 123,825 | 1,155 | 15,642 |
| 2020 Q4 | 25,839 | 264 | 2,204 |
| Post-EUA (2021 Q1–2025 Q4) | 1,198,156 | 26,331 | 83,176 |

*Note: Absolute counts used to decompose rate changes into numerator versus denominator (total reporting) effects. Auditory AE reports increased 22.8-fold vs. 9.7-fold for total reporting and 5.3-fold for dermatologic reports.*
