# Supplementary material for: Temporal changes in auditory adverse event reporting in the U.S. vaccine adverse event reporting system associated with the COVID-19 vaccine rollout: an interrupted time series analysis with negative control evaluation
Source: Front Public Health. 2026 Sep 15;14:1853253. doi: 10.3389/fpubh.2026.1853253 (PMC13619909; doi:10.3389/fpubh.2026.1853253)
Supplement: Supplementary file 2 [file Data_Sheet_2.DOCX]

## **Supplementary Table 8. Non-COVID-19 vaccine ITS analysis: segmented linear regression with Newey-West standard errors**

| Model | β | 95% CI | p-value |
| --- | --- | --- | --- |
| Linear rate (per 100,000) | 702.4 | 354.3 to 1050.5 | <0.001 |

**Notes:** This analysis was restricted to VAERS reports that did not involve any COVID-19 vaccine (i.e., reports with no VAX_TYPE code beginning with “COVID19”; n = 315,568; auditory AE = 2,776). The model was fitted with Newey-West standard errors (lag = 3) to account for potential autocorrelation. CI = confidence interval; EUA = Emergency Use Authorization; ITS = interrupted time series; VAERS = Vaccine Adverse Event Reporting System.

## **Supplementary Table 9. Non-COVID-19 vaccine ITS analysis: count-based models with offset for total reporting**

| Model | Effect measure | Estimate | 95% CI | p-value |
| --- | --- | --- | --- | --- |
| Quasi-Poisson | IRR | 2.24 | 1.53 to 3.29 | <0.001 |
| Negative binomial | IRR | 2.19 | 1.51 to 3.18 | <0.001 |

**Notes:** This analysis was restricted to VAERS reports that did not involve any COVID-19 vaccine (n = 315,568; auditory AE = 2,776). Both models included an offset for the log-transformed total number of reports per quarter to account for changes in the reporting denominator. IRR = incidence rate ratio; CI = confidence interval; ITS = interrupted time series; VAERS = Vaccine Adverse Event Reporting System.

## **Supplementary Table 10. Non-COVID-19 vaccine ITS analysis: age-stratified immediate intervention effects**

| Age group | β | 95% CI | Raw p-value | BH-adjusted p-value |
| --- | --- | --- | --- | --- |
| <18 years | -404.1 | -621.1 to -187.2 | 0.001 | 0.001 |
| 18–64 years | 1391.7 | 865.5 to 1917.9 | <0.001 | <0.001 |
| ≥65 years | 1351.7 | 807.8 to 1895.5 | <0.001 | <0.001 |

**Notes:** This analysis was restricted to VAERS reports that did not involve any COVID-19 vaccine. The β coefficient represents the immediate change in auditory AE reporting rate (per 100,000 reports) following the EUA in 2020 Q4, estimated from separate segmented linear regression models fitted within each age group with Newey-West standard errors (lag = 3). BH-adjusted p-values were calculated using the Benjamini–Hochberg procedure to control the false discovery rate across the three age comparisons. CI = confidence interval; EUA = Emergency Use Authorization; ITS = interrupted time series; VAERS = Vaccine Adverse Event Reporting System.

## **Supplementary Table 11. Non-COVID-19 vaccine ITS analysis: dermatologic reporting-propensity sentinel (quasi-Poisson count model)**

| Model | Effect measure | Estimate | 95% CI | p-value |
| --- | --- | --- | --- | --- |
| Dermatologic count | IRR | 0.46 | 0.34 to 0.62 | <0.001 |

**Notes:** This analysis was restricted to VAERS reports that did not involve any COVID-19 vaccine. The model used a quasi-Poisson distribution with an offset for the log-transformed total number of reports per quarter. Dermatologic events (“rash” and “dermatitis”) served as a reporting-propensity sentinel to detect non-specific changes in reporting behavior. IRR = incidence rate ratio; CI = confidence interval; ITS = interrupted time series; VAERS = Vaccine Adverse Event Reporting System.
